# Multi-center evaluation of radiomics and deep learning to stratify malignancy risk of IPMNs

**DOI:** 10.1007/s00261-025-05371-3

**Published:** 2026-01-12

**Authors:** Andrea M Bejar, Maria Jaramillo Gonzalez, Ziliang Hong, Gorkem Durak, Elif Keles, Halil Ertugrul Aktas, Zheyuan Zhang, Hongyi Pan, Zeynep Sue Jozwiak, Fergan Bol, Lili Zhao, Chao Chen, Concetto Spampinato, Alpay Medetalibeyoglu, Sukru Mehmet Erturk, Gulbiz Dagoglu Kartal, Yury Velichko, Emil Agarunov, Ziyue Xu, Sachin Jambawalikar, Ivo G Schoots, Marco J Bruno, Chenchan Huang, Tamas Gonda, Candice Bolan, Frank H Miller, Michael B Wallace, Rajesh N Keswani, Pallavi Tiwari, Ulas Bagci

**Affiliations:** 1 Machine & Hybrid Intelligence Lab, Department of Radiology, Northwestern University, Chicago, United States; 2 Department of BiomedicalEngineering, University of Wisconsin–Madison, Madison, United States; 3 Department of Radiology, Bakirkoy Dr. Sadi Konuk Research And Training Hospital, Istanbul, Turkey; 4 Department of Preventive Medicine (Biostatistics), Northwestern University, Chicago, United States; 5 Department of Biomedical Informatics, Stony Brook University Hospital, Stony Brook, United States; 6 University of Catania, Catania, Italy; 7 Department of Internal Medicine, Istanbul University, Istanbul, Turkey; 8 Department of Radiology, Istanbul University, Istanbul, Turkey; 9 Division of Gastroenterology and Hepatology, New York University, New York, United States; 10 Nvidia (United States), Bethesda, United States; 11 Department of Radiology, Columbia University, New York, United States; 12 Department of Radiology and Nuclear Medicine, Erasmus MC, Rotterdam, Netherlands; 13 Departments of Gastroenterology and Hepatology, Erasmus MC, Rotterdam, Netherlands; 14 Department of Radiology, New York University, New York, United States; 15 Department of Radiology, Mayo Clinic, Jacksonville, United States; 16 Departments of Radiology, Biomedical Engineering, Medical Physics, University of Wisconsin–Madison, Madison, United States; 17 William S. Middleton Memorial Veterans Hospital, Madison, United States

**Keywords:** Radiomics, Deep learning, Pancreatic intraductal neoplasms, Artificial intelligence, Pancreatic cyst, Magnetic resonance imaging

## Abstract

**Purpose:**

Distinguishing high-risk intraductal papillary mucinous neoplasms (IPMNs) from low-risk lesions remains a clinical challenge, often resulting in unnecessary procedures due to limited specificity of current methods. While radiomics and deep learning (DL) have been explored for pancreatic cancer, cyst-level malignancy risk stratification of IPMNs remains untapped.

**Methods:**

Our multi-institutional assessed the feasibility of AI for predicting IPMN dysplasia grade using cyst-level image features using 359 T2-weighted (T2W) MRI images from seven centers. We developed and compared 2D and 3D radiomics-only, DL-only, and radiomics-DL fusion models using expert radiologist scoring as a baseline reference. Model performance was evaluated using held-out test data.

**Results:**

The radiomics-DL fusion model showed the highest discriminatory ability on the test set AUC of 69.2%, outperforming the radiomics-only model, AUC of 66.5%. Expert accuracy varied widely from 37.4% to 66.7%, and the inter-rater agreement varied as well with weighted Cohen’s kappa coefficients of 0.33–0.67.

**Conclusion:**

The fusion model, which combines DL with radiomics features from routine T2W MRI, shows promise for objective, cyst-level risk stratification of IPMNs in a multi-center cohort, outperforming radiomics-only models and nearly matching expert radiologists using only T2W and T1-weighted (T1W) sequences. While performance requires improvement for standalone clinical use, this approach offers a scalable, non-invasive method to potentially improve diagnostic accuracy and reduce unnecessary surgical interventions.

**Supplementary Information:**

The online version contains supplementary material available at 10.1007/s00261-025-05371-3.

## Introduction

The increasing detection of *pancreatic cysts* has become a significant clinical challenge [1–3]. A substantial burden is imposed on patients from invasive procedures and surgical risks, and on healthcare systems with significant costs associated with repeat surveillance and intervention. Intraductal papillary mucinous neoplasms (IPMNs) are a potentially premalignant cyst subtype that constitute 50–80% of incidental pancreatic lesions [1–3]. Despite their premalignant nature, the risk of malignant transformation in IPMNs remains poorly defined. Resection studies report a wide range of malignancy rates: 1–38% for branch duct (BD) IPMN and 33–85% for main duct (MD) IPMN. Current rates are likely overestimating the true rate of progression due to selection biases from resection studies [2, 3]. The most recent international consensus guidelines, the *2023 Kyoto Criteria*, represent the current standard for IPMN management [2]. However, existing guidelines contribute to patients and healthcare system burden due to limitations in their risk assessment accuracy. These shortcomings frequently lead to invasive diagnostic procedures and high-risk surgical resections, especially for lesions ultimately deemed low-grade [4–9].

Magnetic resonance imaging (MRI) and endoscopic ultrasound (EUS) with fine needle aspiration (FNA) are the primary modalities for IPMN diagnosis and characterization [2]. EUS-FNA is particularly valuable for evaluating pancreatic cysts and assessing the degree of dysplasia. Accurate pre-procedural characterization is essential to reduce patient burden and costs. Although EUS-FNA procedures carry a low complication rate (up to 3%) and rare mortality, the diagnostic sensitivity is limited, ranging from 4.8% to 61.6% [10–12]. Thus, this invasive, operator-dependent, and costly procedure still cannot reliably exclude malignancy and leaves patients at risk of bleeding, pancreatitis, and infection [2, 6, 10, 11]. A leading indication for the surgical resection of cystic lesions is a concern for malignancy [13]. However, pancreatic resections are major surgeries with significant morbidity and mortality rates and as a result it is critical to diagnose suspicious lesions prior to surgery [14, 15].

Radiomics, leveraging high-throughput quantitative image analysis, enables the extraction and analysis of quantitative features imperceptible to the human eye [16]. Deep learning (DL), a neural network-based advanced artificial intelligence (AI) technique, utilizes convolution to effectively extract and discern complex imaging patterns [17]. While radiomics and DL have advanced pancreatic tumor detection and segmentation in computed tomography (CT) and MRI, their application to characterization of premalignant lesions including IPMNs remains nascent [18]. Recent studies propose radiomics, DL, or fused models for IPMN diagnosis and classification [19–23]. However, critical barriers persist. The pancreas’ retroperitoneal anatomy and heterogeneous parenchyma complicate image analysis, and IPMNs exhibit marked variability in morphology and texture [18]. Additionally, DL demands large, diverse datasets, yet pancreatic MRI—the optimal and preferred modality for cyst characterization—remains scarce and protocol-dependent [3, 24–26]. Our work aims to address these gaps by performing a large, multi-center evaluation on cyst regional level features from T2-weighted (T2W) MRI of MD- and mixed-type IPMNs, to predict dysplasia grade using 2D and 3D radiomics, DL, and fusion approaches.

## Methods

### Data collection and subject selection

**Fig. 1:**
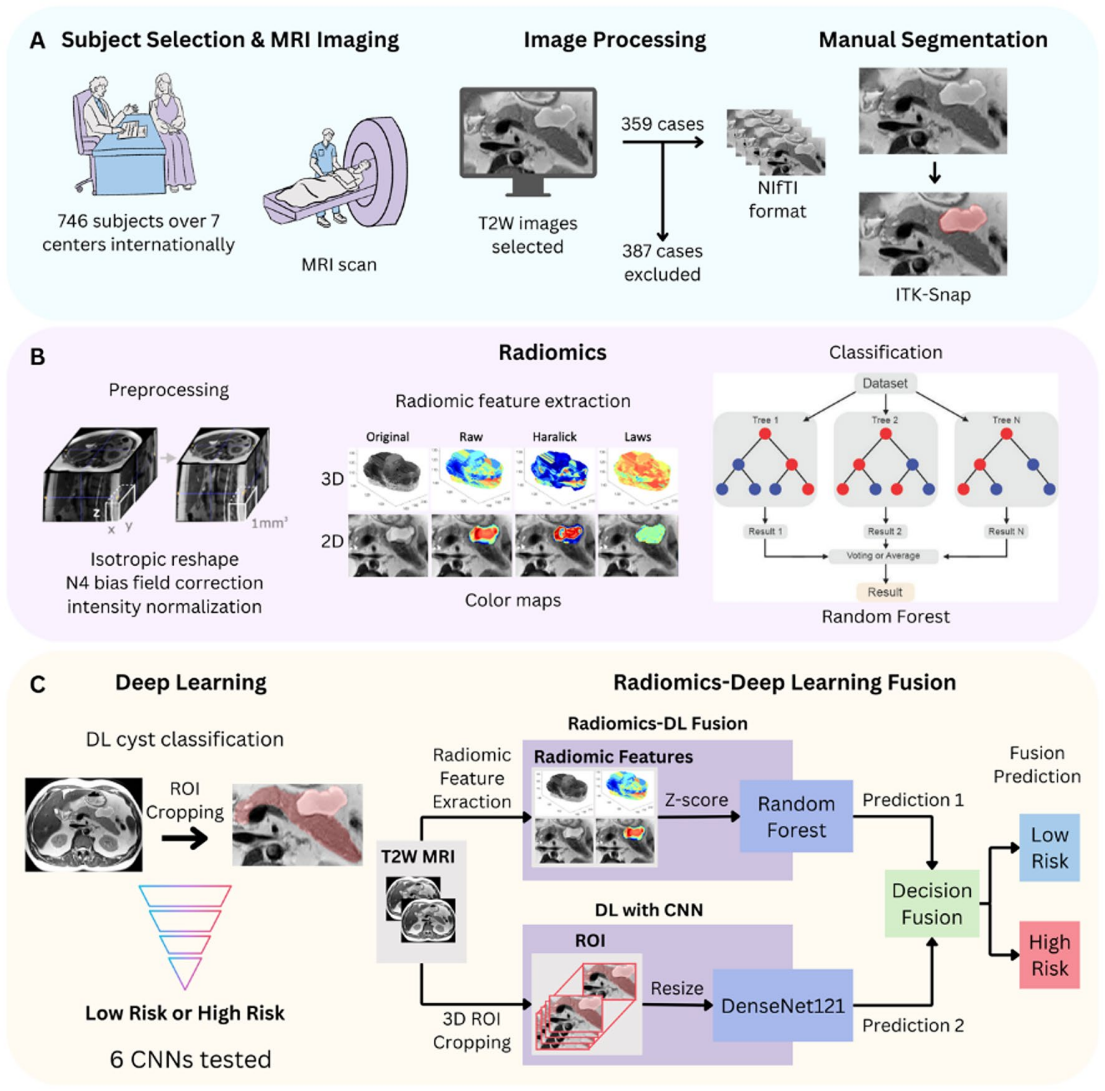
Diagram of patient selection, data set curation, and radiomics and deep learning (DL) pipelines. **A** 746 patients were selected and received MRI imaging from seven centers between three countries; images were then preprocessed and manually segmented. **B** 2D and 3D radiomic features were extracted and classified using a random forest algorithm. **C** A DL-only analysis was conducted, then we developed a radiomics-DL fusion algorithm [27]

**Fig. 2:**
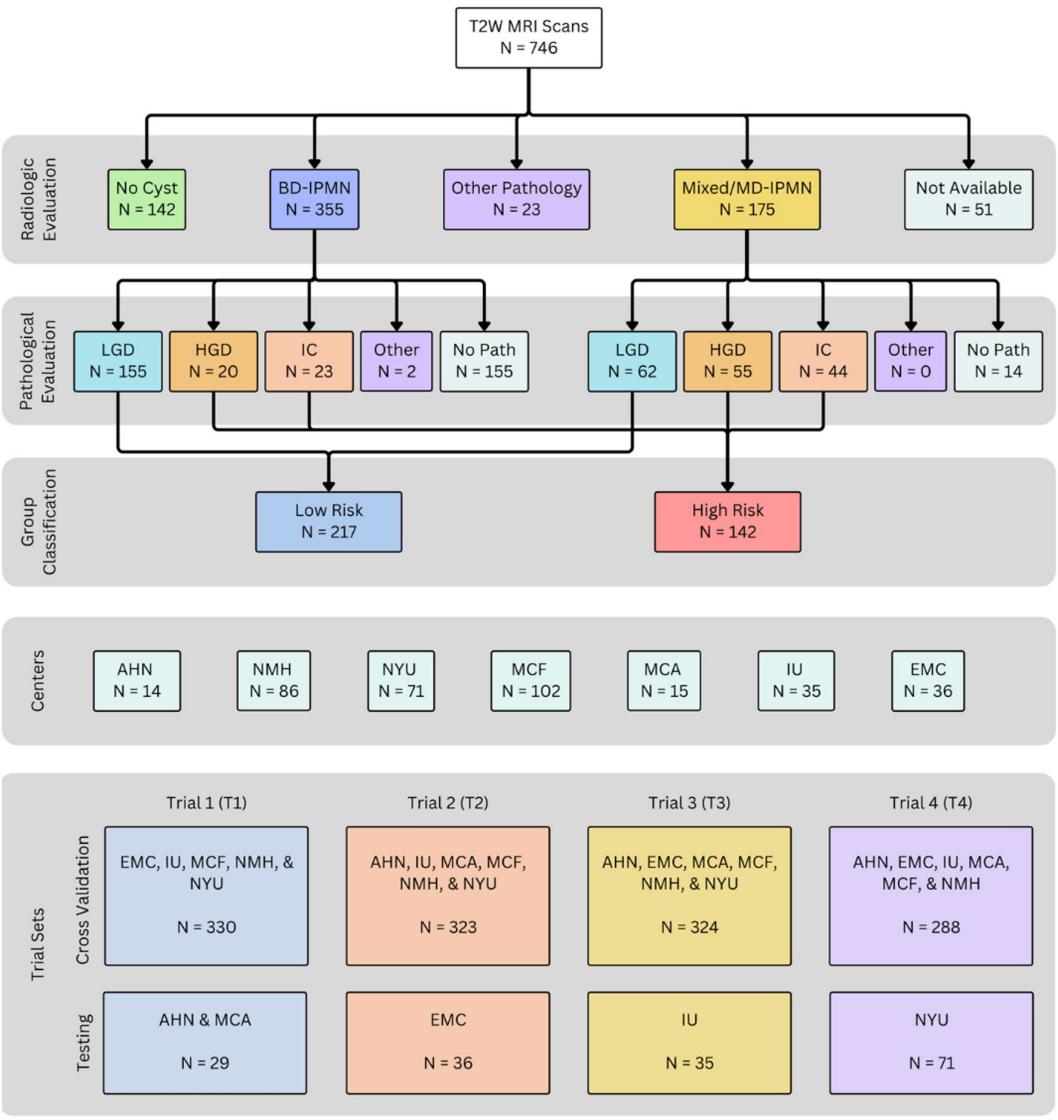
Flowchart of the subject selection and classification. Bottom panel provides breakdown of centers used for testing and cross validation of the four trial sets used in the radiomics, DL, and radiomics-DL fusion analyses

Our retrospective study was approved by an Institutional Review Board (IRB), and all images were de-identified prior to usage in accordance with ethical standards. Figure 1 provides an overview of the study design. We collected 746 T2W MRI scans from patients over 18 years of age undergoing assessment for pancreatic cystic lesions between March 2004 and June 2024. Scans were collected from seven centers: Allegheny Health Network (AHN), Erasmus Medical Center (EMC), Istanbul University (IU) Hospital, Mayo Clinic Florida (MCF), Mayo Clinic Arizona (MCA), Northwestern Memorial Hospital (NMH), and New York University Langone Hospital (NYU) (Fig. 1-A). From an initial cohort of 746 subjects, 359 met these inclusion criteria and were selected for analysis (Fig. 2). The selected cohort had a mean age of 67.2 ± 10.8 years and was 53% female. Images were acquired on Siemens, Philips, or GE scanners with either 1.5 T or 3 T field strength. After collection, images were selected converted to Neuroimaging Informatics Technology Initiative (NIfTI) format for analysis. We selected axial, non-fat-suppressed T2W scans. Slice thicknesses of original Digital Imaging and Communications in Medicine (DICOM) files were between 3 and 8 mm and voxel heights of the converted NIFTI files were 3–15.9 mm. This data set includes abdominal MRIs of subjects with pancreatic cysts that were selected from an extended version of the *PanSegNet dataset* by our multi-center group (Zhang et al., 2025) [25].

We evaluated the radiologic and histopathology results of all subjects prior to inclusion in our study (Fig. 2). On radiologic evaluation, 216 scans were excluded due to the absence of a pancreatic cyst, the presence of a different histopathology, or an unavailable radiologic result. Histopathology of the remaining 530 was evaluated via EUS-FNA or surgical resection. Among this cohort, 171 either did not undergo intervention or had histopathology findings negative for IPMN, leaving 359 patients for our study.

## Subject classification

Dysplasia grades for BD-IPMN were determined via histopathology from EUS-FNA or surgical resection. All MD or Mixed IPMN were surgically resected and histopathologically evaluated. Subjects were then grouped based on dysplasia grade: lesions with low grade dysplasia (LGD) as Low Risk, and lesions with high grade dysplasia (HGD) and/or invasive carcinoma (IC) as High Risk (Table 1). The Low-Risk group was composed of 217 scans, of which 78.2% were BD-IPMN and 38.5% were MD or mixed-type IPMN. The High-Risk group included 142 subjects in total, 27.7% of which were BD-IPMN and 61.5% were MD or mixed-type IPMN cases.

**Table 1:** Breakdown of IPMN subtype in the low and high-risk groups

	BD	Mixed/Main	Total
Low Risk (%)	155 (71.4)	62 (28.6)	217
High Risk (%)	43 (30.3)	99 (69.7)	142

## Image quality assessment

To evaluate center variabilities caused by imaging devices and acquisition protocols, a total of 21 image quality indicators were calculated using the open-source MRQy tool [28]. Quality indicators included statistical values of intensities (e.g. mean, range, variance) and second-order statistics or filter-based measures (e.g. contrast per pixel, entropy focus criterion, and signal-to-noise ratios). The values of these indicators were then projected into a 2D plot using Uniform Manifold Approximation and Projection (UMAP) for Dimension Reduction. Before UMAP projection, each feature was normalized across the dataset using three different methods: z-score, minmax, and data whitening.

## Manual segmentation

The index lesion for each MRI scan was segmented manually using ITK-Snap (Version 4.2.0) and reviewed by an interdisciplinary team including radiologists [29]. All segmentation masks were then reviewed a second time by two radiologists (GD, an abdominal radiologist with seven years of experience, and FB, a general radiologist with four years of abdominal radiology experience) to ensure accuracy (GD, FB, YBT). For subjects with BD-IPMN, the index lesion was defined as the cyst that was sampled in EUS-FNA or that was surgically resected. For mixed and MD-IPMN subjects, all regions with cystic involvement were surgically resected. Approximated MD cyst boundaries were discussed between abdominal radiologists and decided in consensus prior to segmentation due to the complex nature of their anatomy. Concomitant cystic lesions were not included in the analyses.

Interobserver and intraobserver agreements were assessed to evaluate the quality and reproducibility of image segmentations. These are evaluated by calculating the Dice Similarity Coefficient (DSC) and Hausdorff distance (HD95) for each. To assess interobserver agreement, 30 MRI scans were selected randomly from the cohort and were segmented by one of the expert radiologists. These segmentations were compared against existing segmentations. To determine intraobserver agreement, 20 randomly selected MRI scans were segmented a second time after a wash-out period of two weeks.

Radiomics and DL methods are discussed below. The data from each center was grouped into four trial sets for testing while data from the remaining centers is used for cross-validation (Fig. 2). In order to simulate a real scenario, the test set consists of total data from one or two centers in each trial, whichresults in a different amount of test set data in each trial. These sets are referred to as Trial 1 (T1), Trial 2 (T2), Trial 3 (T3), and Trial 4 (T4). The groupings of studies across Trials were chosen to balance the representation of low-, and high-risk studies across the training and test sets. To ensure robust and comprehensive validation, models from each trial were evaluated using a different center’s data for testing.

## Radiomics-only analysis

We conducted radiomics-only analyses using either 2D or 3D radiomic features (Fig. 1B). Images were resized to achieve an isotropic voxel size of 1 mm^3^ (3D analysis) or an isotropic pixel size of 1 mm^2^ (2D analysis) using linear interpolation. An N4 bias field correction was applied to reduce low-frequency variations in the acquired signals [30]. Intensity values were normalized using the min-max normalization technique [31]. Then, radiomic features were extracted from the preprocessed images using in-house software and the *collageradiomics* Python package [32–35]. For the 3D analysis, 763 radiomic features were extracted from the entire volume of the cyst. For the 2D analysis, 447 features were extracted from axial plane slices. The window sizes used to construct the Gray-Level Co-occurrence Matrices (GLCM) were w = 3 × 3, w = 5 × 5, and w = 7 × 7; and the number of gray levels was set to 4, 8, 16, 32, and 64. Selected features and descriptions of radiomic feature families are provided in Supplemental Tables S1 and S2. The features were calculated inside the entire region of interest (ROI) and then each feature was represented by four statistical measures: median, standard deviation, skewness, and kurtosis. A Spearman correlation threshold of 0.6 was applied to remove the most correlated features using the training set of each trial. A 5-fold cross-validation scheme with 50 iterations was then applied to select the best features using a Maximum Relevance Minimum Redundancy (mRMR) algorithm [36]. After cross-validation, features selected in at least 70% of the iterations were selected. A Random Forest (RF) model was trained with the entire training set and tested with the hold-out set.

### Deep learning-only analysis

The DL experiment was done in two parts (Fig. 1-C). First, we applied 5-fold cross-validation on the entire dataset from all centers to select the best performing DL architecture that would then be utilized in the fusion experiment. We assessed the performance of six advanced convolutional neural networks (CNNs): EfficientNet-B0 [37], MobileNet-V2 [38], ResNet-34 [39], ResNet-50 [39], ShuffleNet-V2 [40], and DenseNet-121 [27]. ROIs were cropped based on whole pancreas segmentations published in Zhang et al., 2025 [25]. Images were shuffled and resized to 96 × 96 × 96 for training. Models were trained for a total of 200 epochs using stochastic gradient descent (SGD) with a momentum of 0.9 and a batch size of 2. The initial learning rate was set at 0.001 and decreased by a factor of 10 for every 30 epochs. A 5-fold cross-validation process was applied to enhance result robustness. In the second part, we split the dataset by center, based on the four trial sets as described in Fig. 2. Each set was then used to retrain the best performing DL architecture from part one using the same hyperparameters and training strategies, each model was trained from scratch.

## Radiomics-deep learning fusion algorithm

Our radiomics-DL fusion algorithm was developed by fusing decision probabilities of the radiomics RF classifier with the best performing CNN in the DL-only analysis, DenseNet121 [27]. A visualization of our fusion pipeline is provided in Fig. 1-C. Radiomics feature refinement was done by applying a 5-fold cross-validation and selecting radiomic features with a Spearman correlation coefficient below 0.6 to minimize redundancy. Both the radiomics-based RF model and the DL model were retrained using the same training set, which was consistently split to ensure comparability. After training, the predicted probabilities from both models were fused, and the combined output was evaluated on the validation and test sets. For decision-level fusion, the probability outputs from both the DenseNet121 and RF models were combined. Inspired by our earlier work, Yao et al. 2023 [23], we applied an exact sample to the fusion method and found the best hyperparameter with grid search on fivefold cross validation [18]. Two hyperparameters were introduced in the fusion method: the threshold *t* and the weight *k*. If the radiomics prediction exceeds the threshold *t*, the final model output was solely based on the radiomics prediction, and the DL prediction was discarded. Otherwise, the fusion output was a weighted combination of the predictions from the radiomics-based model and the DL model, with the weight of the radiomics-based model set to *1-k* and weight of the DL model set to *k*. The fusion pipeline was conducted twice, using either 2D or 3D radiomic features. Weighted averages were calculated because of differences in the number of subjects represented in each center.

## Radiologist visual scoring

Images were visually scored by three expert radiologists [GD, FB, YBT] using the imaging features of the Kyoto Criteria [2]. Cysts were given the label of No Risk, Low Risk, or High Risk according to radiological assessment. Before the evaluation, radiologists were instructed to classify the cysts based on the radiologic features outlined in the Kyoto guidelines. Aside from this, no additional detailed guidance was provided, and they were asked to follow their own interpretations as closely as possible, just as they would in real life. Any inaccuracies in scoring that might result from this approach are described in the limitations section. The radiologists were not told that the cysts were confirmed IPMN to emulate real-life, initial cystic lesion evaluation. A third, “no risk,” class was included to avoid biasing scores because radiologists often cannot confirm that cystic lesions are IPMNs in the clinical setting. Additionally, scorers were blinded to subject clinical information and histopathological outcome, utilized only T2W and contrast-enhanced T1-weighted (T1W) sequences, and did not have access to previous imaging. 13 cases from the study cohort were excluded from this analysis because T1W images were not available (*n* = 347). A pairwise assessment of weighted kappa statistics was calculated to evaluate agreement between raters. Sensitivity and specificity were calculated to evaluate the accuracy that the radiologists identified a high-risk lesion correctly.

## Results

In this section, we evaluate the development and performance of three advanced machine learning algorithms for the stratification of IPMN dysplasia grade in MRI: (1) radiomics-only; (2) DL-only; (3) radiomics-DL fusion. Each approach was assessed using rigorous validation protocols across our multi-center dataset.

### Evaluation of dataset heterogeneity using UMAP

**Fig. 3:**
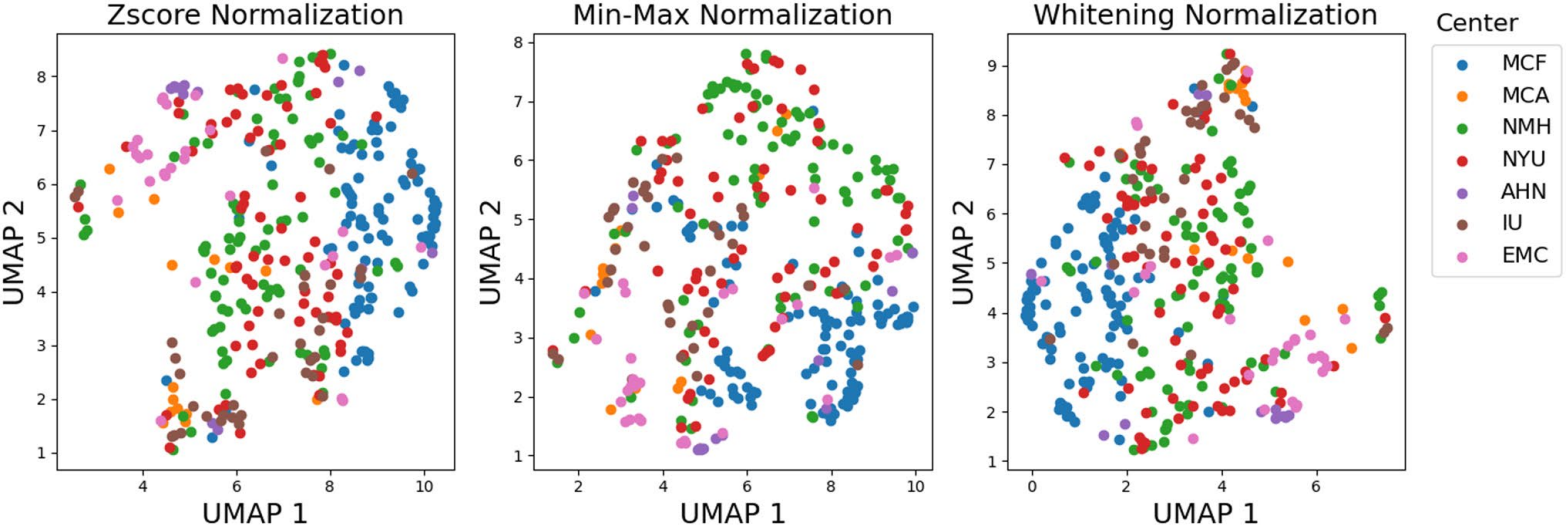
UMAP of quality indicators (projected into x and y axes from 21 quality indicators) per center using different normalization methods. Centers: Mayo Clinic Florida (MCF), Mayo Clinic Arizona (MCA), Northwestern Memorial Hospitals (NMH), New York University (NYU), Allegheny Health Network (AHN), Istanbul University (IU) Hospital, and Erasmus Medical Center (EMC)

UMAP analysis revealed distinct clusters of scans associated with each center that had a close association to MRI sequence voxel height (Fig. 3). One cluster comprised scans primarily from MCF (blue), characterized by a mean voxel height of 4 mm. Another cluster included scans from the NMH and NYU (green and red), with respective mean voxel heights of 5.5 mm and 5 mm. A separate cluster consisted of EMC scans (pink), notable for a 7.3 mm mean voxel height. Scans from the MCA, AHN, and IU (orange, purple, and brown) had a 7 mm mean voxel height and were distributed outside these primary clusters.

### Manualsegmentation of index cystic lesions

**Fig. 4:**
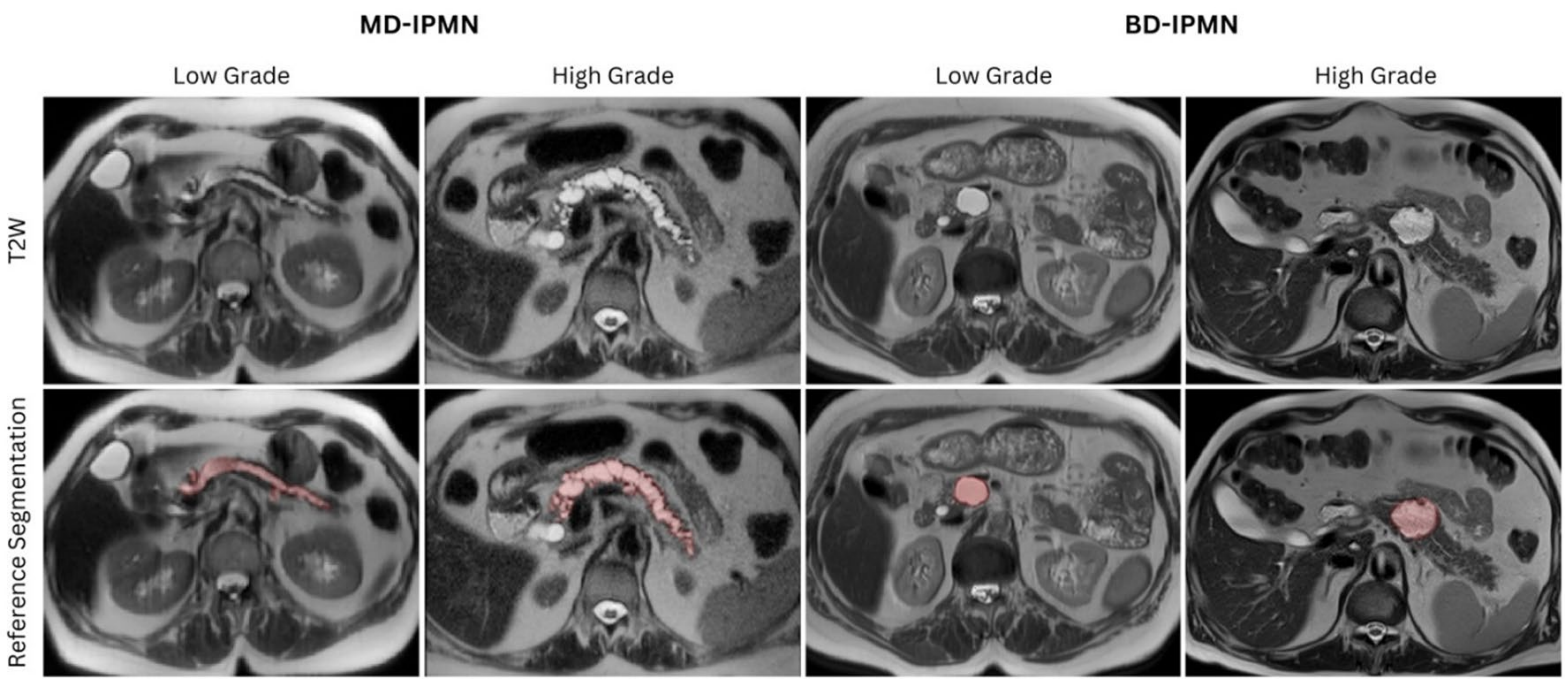
Representative T2-weighted (T2W) images and reference segmentations of high-grade and low-grade IPMNs. The first row shows T2W MRI images, and the second row shows reference segmentations of high-grade and low-grade, main-duct (MD)-IPMN and branch-duct (BD)-IPMN cases

Intraobserver mean DSC across three readers (GK, AMB, and HEA) was 80% and a HD95 of 6.63 mm. Interobserver mean DSC was 75% with a HD95 of 7.2 mm. Representative T2W MRI images and corresponding segmentations of MD and BD-IPMN across varying dysplasia grades are shown in Fig. 4.

### Visual scoring and risk prediction

Diagnostic performance metrics are summarized in Table 2. Sensitivity analysis revealed that Rater 3 achieved the highest detection rate, 72.3% (95% CI: 64.1–79.5%), while Rater 1 demonstrated superior specificity of 64.5% (95% CI: 57.8–70.9%). In cases with concordant majority readings (*n* = 337), positive percent agreement was calculated. Pairwise comparisons of overall accuracy demonstrated significant heterogeneity, with the highest concordance observed being 80.1% (*p* < 0.001) between Raters 1 and 3, and the lowest being 48.1% (*p* = 0.042) between Raters 1 and 2. Cohen’s kappa coefficient analysis indicated fair to moderate agreement between raters with κ of 0.33 to 0.67, as detailed in Table 2. The inter-observer variability demonstrated statistically significant heterogeneity (Cochran’s Q test, *p* < 0.001).

**Table 2:** Comparison of individual, majority, and **i**nter-rater of performance in visual scoring of IPMN by the imaging features of the Kyoto criteria [2]

	Acc (%)	Sens (%)	Spec (%)
Rater 1	66.7	68.9	64.5
Rater 2	37.4	42.2	32.6
Rater 3	57.1	72.3	41.8
Majority	66.9	70.6	63.2
	Comparison	Acc (95% CI)	Weighted Kappa (95% CI)
Inter-rater	Rater 1 vs. 2	48.1 (42.7, 53.5)	0.33 (0.27, 0.39)
	Rater 2 vs. 3	61.7 (56.3, 66.8)	0.67 (0.59, 0.74)
	Rater 1 vs. 3	80.1 (75.5, 84.1)	0.47 (0.39, 0.53)

Sensitivity (sens) and specificity (spec) of each rater (*n* = 347), and a pooled sensitivity and specificity for subjects that received the same score by a majority of the raters (*n* = 337). Inter-rater performance was assessed with accuracy (acc) and weighted Cohen’s kappa coefficient analysis

### Prediction using 2D and 3D radiomic features

**Fig. 5:**
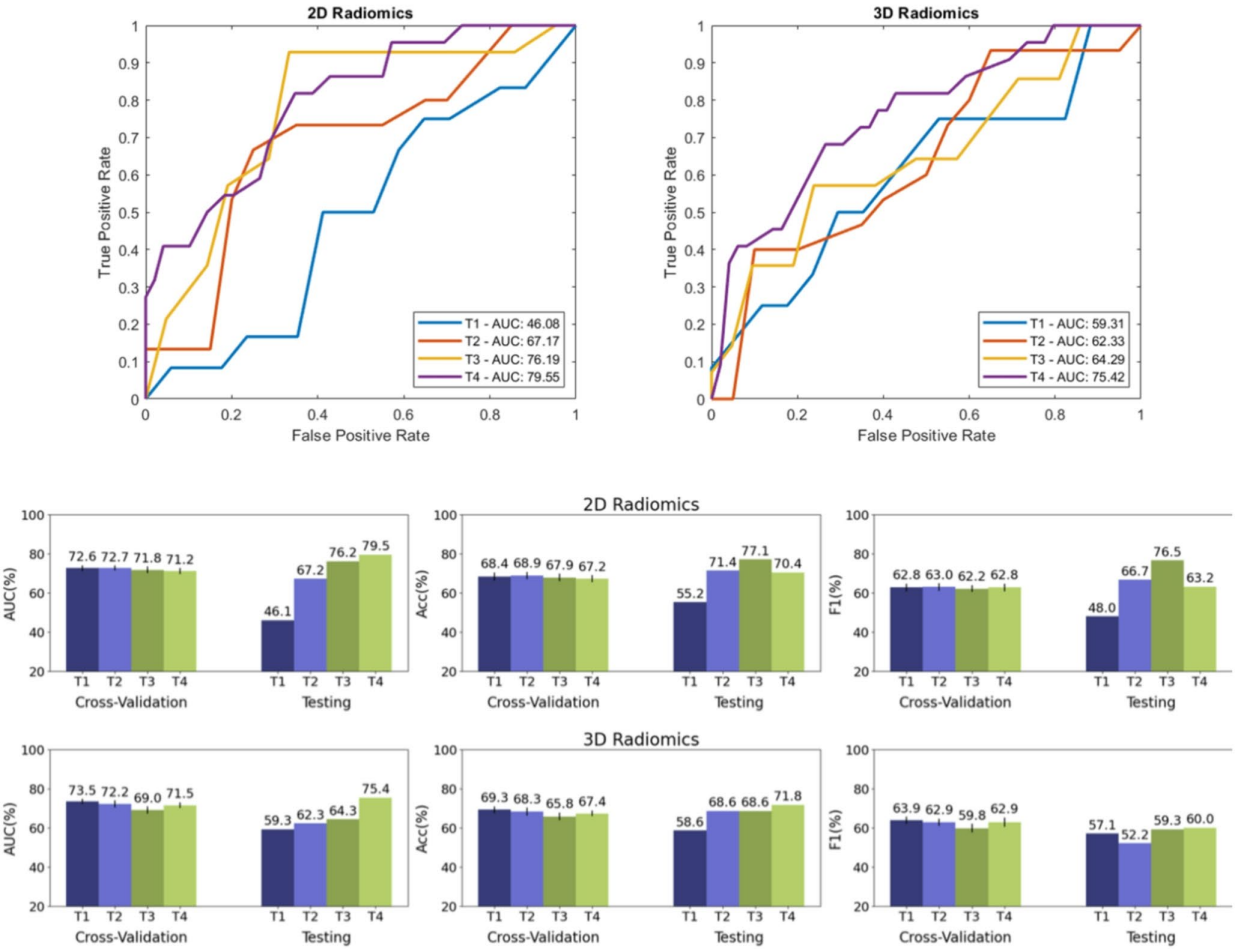
Radiomics-only analysis performance metrics. (Top) Receiver operating characteristic curves of 2D and 3D radiomics predictions distinguishing between Low and High-Risk groups in the testing set. (Bottom) Low vs. High Risk classification comparison in cross-validation set’s mean (%) Area Under the Curve (AUC), accuracy (acc), and F1 for 2D and 3D analyses. Means are written above the error bars, and error bars show standard deviation

The 2D radiomic analysis yielded a mean Area Under the receiver operating characteristic Curve (AUC) of 66.4% with mean accuracy of 65.9%. The 3D analysis yielded a mean AUC of 66.5% and a mean accuracy of 66.1% (Table 3). The corresponding Receiver Operating Characteristic (ROC) curves and bar plots displaying the individual testing set AUC, acc, and F1 are shown in Fig. 5.

**Table 3:**
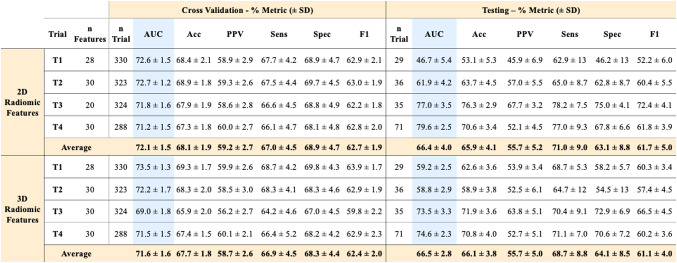
Radiomics-only results for 2D and 3D analysis for each trial set

### Comparing the performance of six DL architectures in predicting IPMN dysplasia grade

Among the tested various CNNs, DenseNet121 [27] demonstrated the highest AUC at 73.3% (Table 4). In comparison, ResNet-34 [39] achieved a slightly lower AUC of 73.1%. Lightweight models, such as EfficientNet-B0 [37] and ShuffleNet-V2 [40], exhibited demonstrably lower AUC values of 68.1% and 66.1%, respectively.

**Table 4:** Deep learning results and standard deviation of IPMN cyst malignancy risk stratification in 5 folds cross-validation [27, 37–40]

	AUC (%)	Acc (%)	Sens (%)	Spec (%)
DenseNet121	73.3 ± 7.9	68.0 ± 7.7	46.5 ± 2.3	82.7 ± 6.5
Mobilenetv2	73.0 ± 2.9	66.6 ± 2.3	N/A	N/A
ResNet34	73.1 ± 4.7	68.5 ± 4.4	N/A	N/A
ResNet50	71.8 ± 6.3	66.0 ± 6.1	N/A	N/A
ShuffleNet-V2	66.6 ± 5.7	61.3 ± 1.7	N/A	N/A
EfficientNet-B0	68.1 ± 1.1	65.6 ± 6.0	N/A	N/A

### Evaluation of 2D and 3D radiomics-DL fusion algorithms

Using 2D radiomic features, the fusion model achieved a weighted average AUC of 74.3% and an accuracy of 71.0% in cross-validation. In independent testing, this 2D feature fusion model yielded an AUC of 69.2% and an accuracy of 61.6% (Table 5). When trained with 3D radiomic features, the fusion model demonstrated a weighted average AUC of 99.7% and an accuracy of 98.4% in cross-validation. In independent testing, it achieved an AUC of 68.3% and an accuracy of 62.7%.

Compared to visual scoring results described in the above section, our radiomics-DL fusion models demonstrated superior performance to two of three radiologists and comparable performance to the majority-voting-based results. The fusion model outperformed two of three individual radiologists in accuracy: 61.6% for 2D fusion, 62.7% for 3D accuracy, 37.4% for Rater 2, and 57.1% for Rater 3. The 2D fusion model achieved higher specificity than all three radiologists: 67.8% for 2D fusion, 64.5% for Rater 1, 32.6% for Rater 2, 41.8% for Rater 3, and 63.2% in cases with majority consensus. It should also be noted that radiologists used both T1W and T2W scans and Kyoto guidelines for determining the cysts stratification while our DL and radiomics analysis used only T2W, indicating the promising and superiority nature of machine generated results.

**Table 5:**
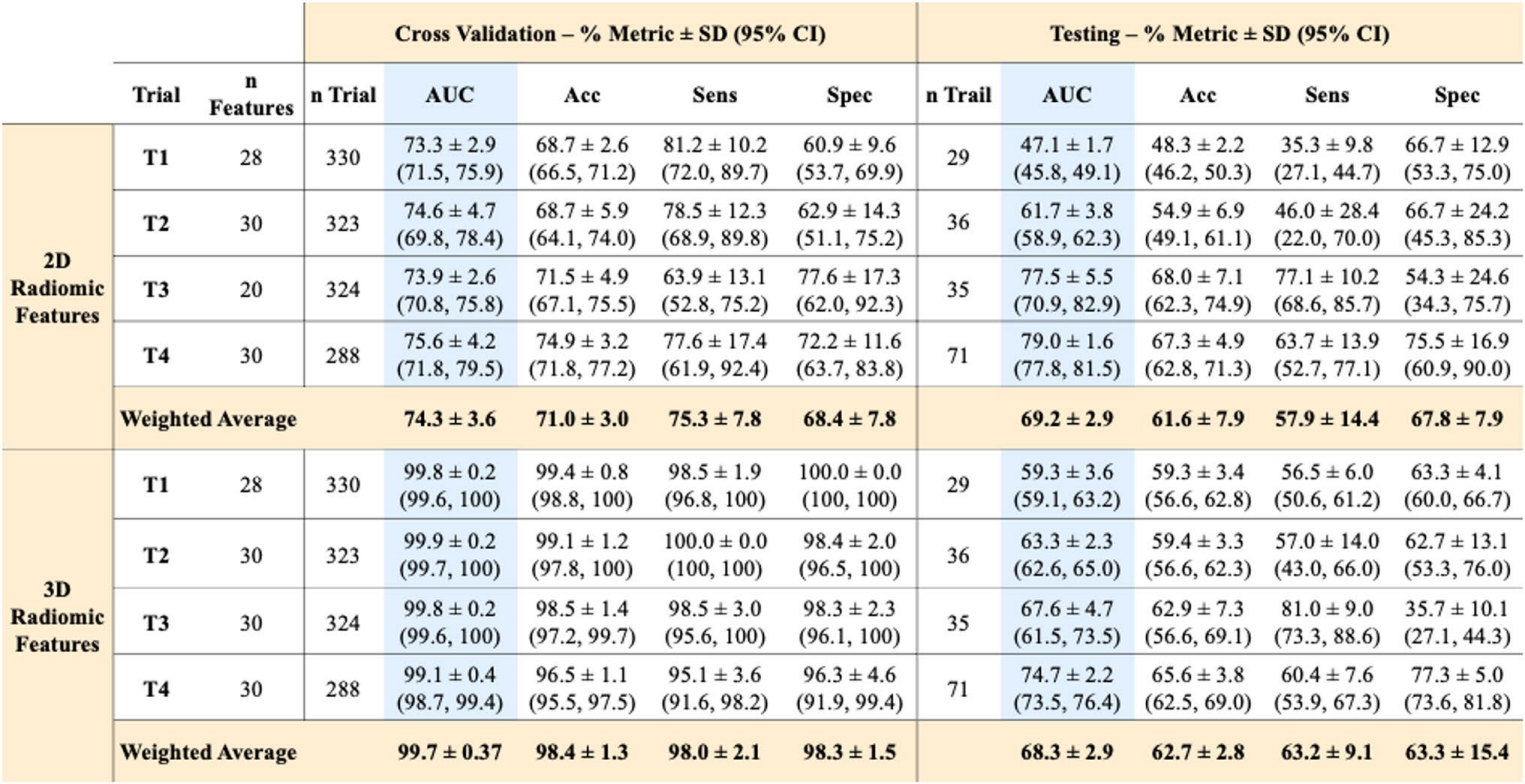
Radiomics-deep learning fusion algorithm results. 2D and 3D radiomic features were fed into DenseNet121 in 5 cross-validation on 4 different trials [27]

## Discussion

In this large multi-center study of 359 subjects, demonstrated the feasibility of radiomics and DL approaches for malignancy risk stratification of IPMN lesions using cyst region–level analysis on T2-weighted MRI. Visual scoring raters had minimal to moderate agreement with weighted Kappa scores of 0.33–0.67 [41]. The visual scoring accuracy for the majority cases and for Rater 1 was similar to the accuracies of the radiomics-only algorithms on the testing set and was higher than the accuracies of the fusion algorithms on the testing set, Rater 2, and Rater 3. Our DenseNet121 based DL-only model achieved the highest performance with an AUC of 73.3% and accuracy of 68.0%), followed closely by our radiomics-DL fusion algorithm using 2D radiomic features with an AUC of 69.2% and accuracy of 61.6% in testing and an AUC of 74.3% and accuracy 71.0% in cross-validation. This performance effectively balanced parameter efficiency and predictive power. Lightweight models, EfficientNet-B0 and ShuffleNet-V2, exhibited lower AUC values, underscoring the trade-off between model complexity and predictive accuracy across diverse architectures. The fusion of DL and radiomics algorithm utilizing 2D radiomic features attained a weighted average AUC of 69.2% and accuracy of 61.6% in testing, and a weighted average AUC of 74.3% and Acc of 71.0% on cross validation. Radiomics-only analyses employing 3D features followed with respective AUC and accuracy of 66.5% and 66.1% on testing. Though, a comparable performance was observed between algorithms utilizing 2D versus 3D radiomics features on the independent test set, a significant discrepancy was observed between cross-validation and independent external testing from 3D radiomics results, suggesting potential overfitting and highlighting the importance of independent-center performance as the clinical meaningful benchmark. This gap likely reflects the relatively small sample size, potential information leakage, and distribution shifts in patient population and imaging protocols across centers. Moreover, the 3D radiomics fusion model contains numerous voxel-level features that are sensitive to protocols and devices’ differences, which may introduce center-specific rather than disease-invariant patterns. These factors together contribute to the underperforming on the independent dataset and emphasize the challenges of model generalization in real-world clinical. It is important to note that the lower performance of T1 could be attributed to lower quality of the sequences from the AHN dataset, which made up a larger proportion of the testing set in this trial. Images from AHN have lower resolution and more artefact which could explain the distribution outside of the primary clusters on UMAP analysis. T1 nevertheless performed well on testing when using 3D radiomic features in the radiomics-only and fusion algorithms with AUCs of 59.2% (SD: 2.5) and 59.3% (95% CI: 59.1, 63.2), respectively. Overall, these advanced methods demonstrated performance that matched or exceeded a limited-input expert radiologist assessment, highlighting their potential to augment clinical decision-making in IPMN management.

In our earlier work (Yao et al. 2023), IPMN malignancy risk was classified using an automatic whole pancreas segmentation algorithm on 246 T1W and T2W MRI scans from five centers [23]. In that work, three algorithms were developed with incorporated clinical features (age, gender, body mass index, diabetes mellitus, and chronic pancreatitis) to accomplish this task: a radiomics-only, DL-only, and DL-radiomics fusion using four CNNs and Vision Transformer (ViT). Algorithms stratified cases as healthy (*n* = 70), low-grade risk (*n* = 85), and high-grade risk (*n* = 91). Our results are not entirely comparable with Yao et al. 2023 [23] because we switched into two class-classification from three-class classification by focusing only in cystic cases. Another key difference compared to our earlier study is our earlier study did not include cyst-type, and all the experiments conducted on a much smaller cohort.

Cui et al. 2021 conducted a study to develop a nomogram to predict the pathological grade of BD-IPMN [42]. The nomogram incorporated clinical features (sex, symptoms, age, CA19-9, and CEA) and radiomic features derived from manually segmented cysts. Their dataset included T2W, T1W, and contrast enhanced T1W scans pertaining to 202 patients collected from three centers. Cases were classified by dysplasia grades as low or high. They found that 24.8% of their BD-IPMN cases had high grade dysplasia. On testing using radiomic-only features, they had specificity, sensitivity, and AUC of 81.6%, 70.0%, and 81.1% respectively on validation. Once radiomic and clinical features were incorporated, their nomogram achieved specificity, sensitivity, and AUC of 79.0%, 90.0%, and 88.4% in validation. While promising, nomograms may perform poorly when applied to populations different from their development cohort, limiting their generalizability across diverse clinical settings [43]. When comparing our studies, their utilization of BD-IPMN only could lead to selection bias because BD-IPMN has a lower risk of malignancy. Additionally, their ratio of high-grade dysplasia cases is lower than ours and may not be representative of a real-world cohort of IPMN which we tried to approximate. Furthermore, authors included an additional scan of contrast enhanced T1W sequences in their analysis while we confined ourselves into conventional T1W and T2W. In comparing our results, their radiomics-only analysis outperformed oursin AUC and specificity. This could be highly likely because their analysis included several clinical features which are known to be predictive of higher risk IPMN [2]. We are aware of the significance of clinical features in predicting IPMN malignancy risk and plan to incorporate them into our future analyses. Despite this, our radiomics-only analysis had similar sensitivity to theirs. This is in spite of our inclusion of multiple centers but could have been due to our larger data set. Overall, the similarities in performance between our two studies suggests that inclusion of clinical markers could augment our algorithm and make it more robust.

To our knowledge, most studies that have used radiomics to classify IPMN are largely CT-based [44–47]. MRI is the preferred imaging method for IPMN classification and monitoring compared to CT because it has no radiation exposure, has higher contrast resolution, and it is better at assessing tissue and cysts [3, 48]. Furthermore, ours is the most comprehensive study on IPMN malignancy risk stratification that utilizes cyst masks in MRI [20, 42, 49]. Cheng et al. 2022 found superior performance of an MRI radiomics algorithm when compared to CT in predicting IPMN malignant potential [20]. Among studies that have utilized MRI, two analyzed only BD-IPMN and the remainder did not specify IPMN type [20, 23, 42, 49]. We found that 38.5% of our Mixed/MD-IPMN and 78.2% of BD-IPMN lesions were Low-Risk. This suggests that many pancreatic resections are unnecessarily performed because a lesion is a MD-IPMN, without any further analysis to stratify lesions that may actually be at risk of malignancy. MD-IPMN is frequently surgically resected in patients that do not have contraindications to surgery, as it has a higher risk of malignant transformation than BD-IPMN [2, 3]. Studies that only include BD-IPMN are excluding an important and under-investigated subtype. We included MD- and mixed-IPMN in our advanced algorithm training to address this gap.

Our study has several limitations that should be considered. Firstly, its retrospective design inherently limits causal inference and introduces potential biases in the historical data collection. The data collected over two decades contributed to variations, including differences in scan quality and uncertainties regarding the accurate grading of dysplasia. The experience levels of operators and pathologists varied across cases, potentially affecting the reliability of dysplasia assessments. Additionally, there was no standardized protocol for selecting cases for EUS-FNA, which may introduce bias since some patients might have undergone EUS for reasons unrelated to the malignancy risk of cystic lesions. Consequently, cytology might have been obtained from cysts that were not classified as high-risk based on imaging. Furthermore, the appearance of cysts may have changed in MRI images taken after the EUS procedure, potentially complicating image analysis. Despite the risk of cyst appearance changes following EUS, we have found our results to be reliable using segmentations of visible cystic lesions. Moreover, our cyst segmentations exhibited a low interobserver variability, DSC of 75%, compared to current standards in the field. However, IPMN can exhibit complex morphology and severe IPMN can cause significant distortion of the normal pancreatic anatomy. Precise and consistent segmentation of these severe cases is challenging. Acknowledging these concerns, we thoroughly reviewed the dataset to ensure its suitability for the study.

An additional limitation results from our exclusive inclusion of sampled cysts. This intentional selection introduced some selection bias. However, focusing on patients at higher risk for malignancy was crucial. EUS-FNA sensitivity is limited and cannot definitively rule out HGD or malignancy, as EUS diagnosed some of our cases. Nevertheless, our observed rates of malignancy risk for BD-IPMNs are similar to higher than those reported in the broader literature, which frequently includes milder cases [2, 3]. Our dataset was collected from seven institutions using various brands of MRI scanners and field strengths (1.5T and 3 T) with differing image acquisition protocols. This variability poses analytical challenges and ultimately affects the algorithm’s performance. This diversity and heterogeneity also strengthens prediction robustness, ensures stability across different environments, and enhances its applicability in real-world clinical settings where imaging protocols frequently vary. Our image analysis was limited to T2W MRI sequences due to data availability constraints. We plan to include and analyze additional MRI sequences in our future studies.

Lastly, radiologist raters utilized only T1W and T2W sequences for expert risk assessment. These sequences alone are insufficient for a thorough visual evaluation and do not fully reflect real-life assessments. Moreover, the radiologist raters lacked access to previous scans, clinical information, or other critical MRI sequences—such as diffusion sequences—that are valuable for accurately estimating risk. These factors could affect the accuracy of visual scoring compared to standard comprehensive imaging analyses. Our study’s limitations ultimately point to several promising directions for future research. Prospective validation studies with standardized imaging protocols would strengthen evidence for clinical translation. Integration of clinical parameters and additional MRI sequences could further improve model performance. Development of ensemble approaches that combine imaging features with other biomarkers (cyst fluid analysis, circulating markers) might provide more comprehensive risk assessment. Finally, extending these methods to predict long-term outcomes rather than cross-sectional histopathology would better align with the clinical goal of identifying lesions likely to progress to malignancy.

In conclusion, our multi-center, pancreatic cyst-focused study demonstrates the feasibility and potential clinical utility of radiomics and DL for IPMN risk stratification using routinely acquired T2W MRI scans. While predictive performance requires further enhancement, our advanced machine learning models achieved performance comparable to, and in some metrics better than, limited-input expert radiologist evaluations in this challenging cohort, offering greater objectivity and reproducibility compared to visual assessment. Given that current international consensus guidelines lack optimal specificity for identifying low-risk IPMNs without invasive procedures, computational tools like ours represent a valuable step toward more precise patient selection for intervention versus surveillance. Hence, our findings have immediate clinical relevance. The fusion model’s comparable performance to expert radiologists, suggests potential for integration into clinical workflows as a decision support tool. By providing objective risk stratification of IPMNs, our approach could reduce the high rates of unnecessary surgical resections of low-risk lesions, particularly for MD-IPMNs which are often resected based solely on morphology. Implementation could take the form of a software plugin for radiology workstations, offering real-time risk assessment during routine reads without disrupting workflow. Cost-effectiveness analyses and prospective validation would be logical next steps toward clinical translation.

## Supplementary Information

Below is the link to the electronic supplementary material.

Supplementary Material 1

## Data Availability

Data Availability: Our MRIs and corresponding excel file for risk status of the patients are available at OSF server (NIH supported data sharing platform) at https://osf.io/74vfs/. CodeAvailability: The underlying code for this study is available in GitHub and can be accessed via this link: https://github.com/Zilian4/IPMN-Radiomics-Plus-Deeplearning.

## References

[1] Schweber AB, Agarunov E, Brooks C, Hur C, Gonda TA (2021) Prevalence, incidence, and risk of progression of asymptomatic pancreatic cysts in large sample real-world data. Pancreas 50(9):1287–92. 10.1097/MPA.0000000000001918 34860813 10.1097/MPA.0000000000001918

[2] Ohtsuka T, Fernandez-del Castillo C, Furukawa T, Hijioka S, Jang J-Y, Lennon AM, et al. (2023) International evidence-based Kyoto guidelines for the management of intraductal papillary mucinous neoplasm of the pancreas. Pancreatology 24(2):225–70. 10.1016/j.pan.2023.12.009 10.1016/j.pan.2023.12.009 38182527

[3] Gonda TA, Cahen DL, Farrell JJ (2024) Pancreatic Cysts. N Engl J Med 391(9):832–43. 10.1056/NEJMra2309041 39231345 10.1056/NEJMra2309041

[4] Heckler M, Michalski CW, Schaefle S, Kaiser J, Büchler MW, Hackert T (2017) The Sendai and Fukuoka consensus criteria for the management of branch duct IPMN-A meta-analysis on their accuracy. Pancreatology 17(2):255–62. 10.1016/j.pan.2017.01.011 28189431 10.1016/j.pan.2017.01.011

[5] Yu S, Takasu N, Watanabe T, Fukumoto T, Okazaki S, Tezuka K, et al. (2017) Validation of the 2012 Fukuoka consensus guideline for intraductal papillary mucinous neoplasm of the pancreas from a single institution experience. Pancreas 46(7):936–42. 10.1097/MPA.0000000000000874 28697135 10.1097/MPA.0000000000000874

[6] Romutis S, Brand R (2023) Burden of new pancreatic cyst diagnosis. Gastrointestinal Endoscopy Clinics 33(3):487–95. 10.1016/j.giec.2023.03.001 37245931 10.1016/j.giec.2023.03.001

[7] Robles EP-C, Maire F, Cros J, Vullierme M-P, Rebours V, Sauvanet A, et al. (2016) Accuracy of 2012 International Consensus Guidelines for the prediction of malignancy of branch-duct intraductal papillary mucinous neoplasms of the pancreas. United European Gastroenterology Journal 4(4):580–6. 10.1177/2050640615623370 27536368 10.1177/2050640615623370 PMC4971792

[8] Bulcke AV, Jaekers J, Topal H, Vanbeckevoort D, Vandecaveye V, Roskams T, et al. (2021) Evaluating the accuracy of three international guidelines in identifying the risk of malignancy in pancreatic cysts: a retrospective analysis of a surgical treated population. Acta gastro-enterologica Belgica 84(3):443–50. 10.51821/84.3.006 34599569 10.51821/84.3.006

[9] Maggi G, Guarneri G, Gasparini G, Fogliati A, Partelli S, Falconi M, et al. (2018) Pancreatic cystic neoplasms: What is the most cost-effective follow-up strategy? Endoscopic Ultrasound 7(5):319–22. 10.4103/eus.eus_44_18 30323161 10.4103/eus.eus_44_18 PMC6199914

[10] Eloubeidi MA, Gress FG, Savides TJ, Wiersema MJ, Kochman ML, Ahmad NA, et al. (2004) Acute pancreatitis after EUS-guided FNA of solid pancreatic masses: a pooled analysis from EUS centers in the United States. Gastrointest Endosc 60(3):385–9. 10.1016/s0016-5107(04)01714-6 15332028 10.1016/s0016-5107(04)01714-6

[11] Polkowski M, Larghi A, Weynand B, Boustière C, Giovannini M, Pujol B, et al. (2012) Learning, techniques, and complications of endoscopic ultrasound (EUS)-guided sampling in gastroenterology: European Society of Gastrointestinal Endoscopy (ESGE) Technical Guideline. Endoscopy 44(02):190–206. 10.1055/s-0031-1291543 22180307 10.1055/s-0031-1291543

[12] Tacelli M, Celsa C, Magro B, Barchiesi M, Barresi L, Capurso G, et al. (2020) Diagnostic performance of endoscopic ultrasound through-the‐needle microforceps biopsy of pancreatic cystic lesions: Systematic review with meta‐analysis. Dig Endosc 32(7):1018–30. 10.1111/den.13626 31912580 10.1111/den.13626

[13] De Pretis N, Mukewar S, Aryal-Khanal A, Bi Y, Takahashi N, Chari S (2017) Pancreatic cysts: diagnostic accuracy and risk of inappropriate resections. Pancreatology 17(2):267–72. 10.1016/j.pan.2017.01.002 28117220 10.1016/j.pan.2017.01.002

[14] Loos M, Al-Saeedi M, Hinz U, Mehrabi A, Schneider M, Berchtold C, et al. (2022) Categorization of differing types of total pancreatectomy. JAMA surgery 157(2):120–8.10.1001/jamasurg.2021.5834 34787667 10.1001/jamasurg.2021.5834 PMC8600456

[15] Collaborative Po (2024) Pancreatic surgery outcomes: multicentre prospective snapshot study in 67 countries. Br J Surg 111(1):znad330. 10.1093/bjs/znad330 38743040 10.1093/bjs/znad330 PMC10771125

[16] Gillies RJ, Kinahan PE, Hricak H (2016) Radiomics: images are more than pictures, they are data. Radiology 278(2):563–77. 10.1148/radiol.2015151169 26579733 10.1148/radiol.2015151169 PMC4734157

[17] LeCun Y, Bengio Y, Hinton G (2015) Deep learning. Nature 521(7553):436–44. 10.1038/nature14539 26017442 10.1038/nature14539

[18] Yao L, Zhang Z, Keles E, Yazici C, Tirkes T, Bagci U (2023) A review of deep learning and radiomics approaches for pancreatic cancer diagnosis from medical imaging. Curr Opin Gastroenterol 39(5):436–47. 10.1097/MOG.0000000000000966 37523001 10.1097/MOG.0000000000000966 PMC10403281

[19] Corral JE, Hussein S, Kandel P, Bolan CW, Bagci U, Wallace MB (2019) Deep learning to classify intraductal papillary mucinous neoplasms using magnetic resonance imaging. Pancreas 48(6):805–10. 10.1097/MPA.0000000000001327 31210661 10.1097/MPA.0000000000001327

[20] Cheng S, Shi H, Lu M, Wang C, Duan S, Xu Q, et al. (2022) Radiomics analysis for predicting malignant potential of intraductal papillary mucinous neoplasms of the pancreas: comparison of CT and MRI. Acad Radiol 29(3):367–75. 10.1016/j.acra.2021.04.013 34112528 10.1016/j.acra.2021.04.013

[21] LaLonde R, Tanner I, Nikiforaki K, Papadakis GZ, Kandel P, Bolan CW, et al. (2019) INN: inflated neural networks for IPMN diagnosis. International Conference on Medical Image Computing and Computer-Assisted Intervention:101-9. 10.1007/978-3-030-32254-0_12 10.1007/978-3-030-32254-0_12 PMC10062388 37011258

[22] Salanitri FP, Bellitto G, Palazzo S, Irmakci I, Wallace M, Bolan C, et al. (2022) Neural transformers for intraductal papillary mucosal neoplasms (IPMN) classification in MRI images. 2022 44th Annual International Conference of the IEEE Engineering in Medicine & Biology Society (EMBC):475-9. 10.1109/EMBC48229.2022.9871547 10.1109/EMBC48229.2022.9871547 PMC9921314 36085787

[23] Yao L, Zhang Z, Demir U, Keles E, Vendrami C, Agarunov E, et al. (2023) Radiomics Boosts Deep Learning Model for IPMN Classification. International Workshop on Machine Learning in Medical Imaging 14349:134 – 43. 10.1007/978-3-031-45676-3_14 10.1007/978-3-031-45676-3_14 PMC10810260 38274402

[24] Zhang Z, Yao L, Keles E, Velichko Y, Bagci U (2023) Deep learning algorithms for pancreas segmentation from radiology scans: A review. Advances in Clinical Radiology 5(1):31–52. 10.1016/j.yacr.2023.05.001

[25] Zhang Z, Keles E, Durak G, Taktak Y, Susladkar O, Gorade V, et al. (2025) Large-scale multi-center CT and MRI segmentation of pancreas with deep learning. Med Image Anal 99:103382. 10.1016/j.media.2024.103382 39541706 10.1016/j.media.2024.103382 PMC11698238

[26] Suman G, Patra A, Korfiatis P, Majumder S, Chari ST, Truty MJ, et al. (2021) Quality gaps in public pancreas imaging datasets: Implications & challenges for AI applications. Pancreatology 21(5):1001–8. 10.1016/j.pan.2021.03.016 33840636 10.1016/j.pan.2021.03.016

[27] Huang G, Liu Z, Van Der Maaten L, Weinberger KQ (2017) Densely connected convolutional networks. Proceedings of the IEEE conference on computer vision and pattern recognition:4700-8. 10.1109/CVPR.2017.243

[28] Sadri AR, Janowczyk A, Zhou R, Verma R, Beig N, Antunes J, et al. (2020) MRQy—An open-source tool for quality control of MR imaging data. Med Phys 47(12):6029–38. 10.1002/mp.14593 33176026 10.1002/mp.14593 PMC8176950

[29] Yushkevich PA, Piven J, Hazlett HC, Smith RG, Ho S, Gee JC, et al. (2006) User-guided 3D active contour segmentation of anatomical structures: significantly improved efficiency and reliability. Neuroimage 31(3):1116–28. 10.1016/j.neuroimage.2006.01.015 16545965 10.1016/j.neuroimage.2006.01.015

[30] Tustison NJ, Avants BB, Cook PA, Zheng Y, Egan A, Yushkevich PA, et al. (2010) N4ITK: improved N3 bias correction. IEEE Trans Med Imaging 29(6):1310–20. 10.1109/tmi.2010.2046908 20378467 10.1109/TMI.2010.2046908 PMC3071855

[31] Bagci U, Udupa JK, Bai L (2010) The influence of intensity standardization on medical image registration. Medical Imaging 2010: Visualization, Image-Guided Procedures, and Modeling 7625:602 – 13. 10.1117/12.843969

[32] Inc T (2022) MATLAB version: 9.13. 0 (R2022b). The MathWorks Inc

[33] Prasanna P, Tiwari P, Madabhushi A (2016) Co-occurrence of local anisotropic gradient orientations (CoLlAGe): a new radiomics descriptor. Sci Rep 6(1):1–14. 10.1038/srep37241 27872484 10.1038/srep37241 PMC5118705

[34] Haralick RM, Shanmugam K, Dinstein IH (1973) Textural features for image classification. IEEE Transactions on systems, man, and cybernetics (6):610–21. 10.1109/TSMC.1973.4309314

[35] Python version: 3.8.2. (2023) Python Software Foundation

[36] Peng H, Long F, Ding C (2005) Feature selection based on mutual information criteria of max-dependency, max-relevance, and min-redundancy. IEEE Transactions on pattern analysis and machine intelligence 27(8):1226–38. 10.1109/TPAMI.2005.159 16119262 10.1109/TPAMI.2005.159

[37] Tan M (2019) Efficientnet: Rethinking model scaling for convolutional neural networks. arXiv preprint arXiv:190511946:6105-14.10.48550/arXiv.1905.11946

[38] Howard AG (2017) Mobilenets: Efficient convolutional neural networks for mobile vision applications. arXiv preprint arXiv:170404861. 10.48550/arXiv.1704.04861

[39] He K, Zhang X, Ren S, Sun J (2016) Deep residual learning for image recognition. Proceedings of the IEEE conference on computer vision and pattern recognition:770-8. 10.1109/CVPR.2016.90

[40] Ma N, Zhang X, Zheng H-T, Sun J (2018) Shufflenet v2: Practical guidelines for efficient cnn architecture design. Proceedings of the European conference on computer vision (ECCV):116 – 31. 10.48550/arXiv.1807.11164

[41] McHugh ML (2012) Interrater reliability: the kappa statistic. Biochemia medica 22(3):276–82 23092060 PMC3900052

[42] Cui S, Tang T, Su Q, Wang Y, Shu Z, Yang W, et al. (2021) Radiomic nomogram based on MRI to predict grade of branching type intraductal papillary mucinous neoplasms of the pancreas: a multicenter study. Cancer Imaging 21:1–13. 10.1186/s40644-021-00395-6 33750453 10.1186/s40644-021-00395-6 PMC7942000

[43] Balachandran VP, Gonen M, Smith JJ, DeMatteo RP (2015) Nomograms in oncology: more than meets the eye. The lancet oncology 16(4):e173-e80. 10.1016/s1470-2045(14)71116-7 25846097 10.1016/S1470-2045(14)71116-7 PMC4465353

[44] Lee DY, Shin J, Kim S, Baek S-E, Lee S, Son N-H, et al. (2024) Radiomics model versus 2017 revised international consensus guidelines for predicting malignant intraductal papillary mucinous neoplasms. Eur Radiol 34(2):1222–31. 10.1007/s00330-023-10158-5 37615762 10.1007/s00330-023-10158-5

[45] Tobaly D, Santinha J, Sartoris R, Dioguardi Burgio M, Matos C, Cros J, et al. (2020) CT-based radiomics analysis to predict malignancy in patients with intraductal papillary mucinous neoplasm (IPMN) of the pancreas. Cancers (Basel) 12(11):3089. 10.3390/cancers12113089 33114028 10.3390/cancers12113089 PMC7690711

[46] Permuth JB, Choi J, Balarunathan Y, Kim J, Chen D-T, Chen L, et al. (2016) Combining radiomic features with a miRNA classifier may improve prediction of malignant pathology for pancreatic intraductal papillary mucinous neoplasms. Oncotarget 7(52):85785. 10.18632/oncotarget.11768 27589689 10.18632/oncotarget.11768 PMC5349874

[47] Lou F, Li M, Chu T, Duan H, Liu H, Zhang J, et al. (2024) Comprehensive analysis of clinical data and radiomic features from contrast enhanced CT for differentiating benign and malignant pancreatic intraductal papillary mucinous neoplasms. Sci Rep 14(1):17218. 10.1038/s41598-024-68067-6 39060387 10.1038/s41598-024-68067-6 PMC11282090

[48] Pozzi-Mucelli RM, Rinta-Kiikka I, Wünsche K, Laukkarinen J, Labori KJ, Ånonsen K, et al. (2017) Pancreatic MRI for the surveillance of cystic neoplasms: comparison of a short with a comprehensive imaging protocol. Eur Radiol 27:41–50. 10.1007/s00330-016-4377-4 27246720 10.1007/s00330-016-4377-4

[49] Flammia F, Innocenti T, Galluzzo A, Danti G, Chiti G, Grazzini G, et al. (2023) Branch duct-intraductal papillary mucinous neoplasms (BD-IPMNs): An MRI-based radiomic model to determine the malignant degeneration potential. Radiol Med 128(4):383–92. 10.1007/s11547-023-01609-6 36826452 10.1007/s11547-023-01609-6

