# Correction to: Multi-center evaluation of radiomics and deep learning to stratify malignancy risk of IPMNs

**DOI:** 10.1007/s00261-026-05466-5

**Published:** 2026-03-28

**Authors:** Andrea M. Bejar, Maria Jaramillo Gonzalez, Ziliang Hong, Gorkem Durak, Elif Keles, Halil Ertugrul Aktas, Zheyuan Zhang, Hongyi Pan, Zeynep Sue Jozwiak, Fergan Bol, Lili Zhao, Chao Chen, Concetto Spampinato, Alpay Medetalibeyoglu, Sukru Mehmet Erturk, Gulbiz Dagoglu Kartal, Yury Velichko, Emil Agarunov, Ziyue Xu, Sachin Jambawalikar, Ivo G. Schoots, Marco J. Bruno, Chenchan Huang, Tamas Gonda, Candice Bolan, Frank H. Miller, Michael B. Wallace, Rajesh N. Keswani, Pallavi Tiwari, Ulas Bagci

**Affiliations:** 1 Machine & Hybrid Intelligence Lab, Department of Radiology, Northwestern University, Chicago, United States; 2 Department of BiomedicalEngineering, University of Wisconsin–Madison, Madison, United States; 3 Department of Radiology, Bakirkoy Dr. Sadi Konuk Research And Training Hospital, Istanbul, Turkey; 4 Department of Preventive Medicine (Biostatistics), Northwestern University, Chicago, United States; 5 Department of Biomedical Informatics, Stony Brook University Hospital, Stony Brook, United States; 6 University of Catania, Catania, Italy; 7 Department of Internal Medicine, Istanbul University, Istanbul, Turkey; 8 Department of Radiology, Istanbul University, Istanbul, Turkey; 9 Department of Radiology, New York University, New York, United States; 10 Nvidia (United States), Bethesda, United States; 11 Department of Radiology, Columbia University, New York, United States; 12 Department of Radiology and Nuclear Medicine, Erasmus MC, Rotterdam, Netherlands; 13 Departments of Gastroenterology and Hepatology, Erasmus MC, Rotterdam, Netherlands; 14 Department of Radiology, Mayo Clinic, Jacksonville, United States; 15 Departments of Radiology, Biomedical Engineering, Medical Physics, University of Wisconsin–Madison, Madison, United States; 16 William S. Middleton Memorial Veterans Hospital, Madison, United States

**Correction to: Abdominal Radiology**

**https://doi.org/10.1007/s00261-025-05371-3**

In the original version of the article, given name of the co-author “Chenchang ” was incorrectly mentioned as “Chen­chang” instead “Chenchan” and 9th affiliation should be corrected from “Division of Gastroenterology and Hepa­tology, New York University, New York, United States” to “Department of Radiology, New York University, New York, United States". The co-author name and 9th affiliation details are corrected. Also, orcid id information is added for the co-author "Chenchan Huang".

The original article has been corrected.

