# Supplementary material for: Multi-center evaluation of radiomics and deep learning to stratify malignancy risk of IPMNs
Source: Abdom Radiol (NY). 2026 Jan 12;51(8):3805–20. doi: 10.1007/s00261-025-05371-3 (PMC13388723; doi:10.1007/s00261-025-05371-3)
Supplement: Supplementary file 1 — Supplementary Material 1 [file 261_2025_5371_MOESM1_ESM.docx]

**SUPPLEMENTAL**

**Supplemental Table S1.** Selected radiomic features across trail sets for 2D and 3D analyses.

|  | **2D** | **3D** |
| --- | --- | --- |
| Trial 1 | 'Collage_skew_InformationMeasureOfCorrelation2_1_nb_8_ws_3' | 'Collage_var_InformationMeasureOfCorrelation2_1_nb_8_ws_5' |
|  | 'Collage_var_InformationMeasureOfCorrelation2_1_nb_16_ws_3' | 'Collage_var_SumEntropy_1_nb_32_ws_7' |
|  | 'Collage_kurt_Contrast_1_nb_16_ws_7' | 'skewness-Haralick info1 ws=5 n=4' |
|  | 'Collage_skew_MaximalCorrelationCoefficient_1_nb_4_ws_3' | 'skewness-Laws S5L5E5' |
|  | 'Collage_kurt_SumAverage_1_nb_16_ws_3' | 'median-Laws E5E5E5' |
|  | 'Collage_kurt_InformationMeasureOfCorrelation2_1_nb_16_ws_3' | 'Collage_skew_DifferenceEntropy_1_nb_32_ws_7' |
|  | 'Collage_kurt_MaximalCorrelationCoefficient_1_nb_4_ws_5' | 'median-Laws R5E5S5' |
|  | 'median-Laws E5L5' | 'median-Laws W5E5S5' |
|  | 'Collage_kurt_SumEntropy_1_nb_16_ws_3' | 'Collage_kurt_InformationMeasureOfCorrelation2_1_nb_4_ws_5' |
|  | 'skewness-Laws S5E5' | 'median-Laws E5L5S5' |
|  | 'kurtosis-Haralick correlation ws=7 n=4' |  |
|  | 'Collage_kurt_InformationMeasureOfCorrelation2_1_nb_64_ws_5' |  |
|  | 'Collage_median_DifferenceVariance_1_nb_64_ws_7' |  |
|  | 'skewness-Laws E5S5' |  |
|  | 'skewness-Gradient sobelxy' |  |
| Trial 2 | 'Collage_kurt_MaximalCorrelationCoefficient_1_nb_4_ws_5' | 'Collage_var_InformationMeasureOfCorrelation2_1_nb_16_ws_3' |
|  | 'median-Laws E5L5' | 'median-Laws E5L5S5' |
|  | 'Collage_skew_InformationMeasureOfCorrelation2_1_nb_8_ws_3' | 'median-Laws E5E5E5' |
|  | 'Collage_var_InformationMeasureOfCorrelation2_1_nb_16_ws_3' | 'skewness-Laws R5R5L5' |
|  | 'skewness-Laws R5S5' | 'median-Laws L3S3L3' |
|  | 'Collage_skew_MaximalCorrelationCoefficient_1_nb_4_ws_3' | 'skewness-Laws S3E3S3' |
|  | 'skewness-Laws S5E5' | 'Collage_kurt_Correlation_2_nb_64_ws_5' |
|  | 'median-Laws W5S5' | 'Collage_kurt_SumAverage_1_nb_32_ws_7' |
|  | 'Collage_kurt_MaximalCorrelationCoefficient_1_nb_4_ws_7' | 'skewness-Laws S5L5E5' |
|  | 'Collage_median_SumEntropy_1_nb_8_ws_3' | 'skewness-Laws S3E3E3' |
|  | 'Collage_kurt_SumEntropy_1_nb_16_ws_5' | 'skewness-Laws W5L5S5' |
|  | 'Collage_skew_SumVariance_1_nb_4_ws_7' | 'kurtosis-Laws L5R5L5' |
| Trial 3 | 'Collage_skew_InformationMeasureOfCorrelation2_1_nb_8_ws_3' | 'skewness-Laws S5W5R5' |
|  | 'skewness-Laws S5E5' | 'skewness-Laws S5L5E5' |
|  | 'skewness-Laws R5S5' | 'median-Laws L3S3L3' |
|  | 'Collage_skew_InformationMeasureOfCorrelation2_1_nb_16_ws_5' | 'median-Laws S3E3E3' |
|  | 'Collage_skew_Correlation_1_nb_8_ws_5' | 'Collage_skew_MaximalCorrelationCoefficient_1_nb_4_ws_5' |
|  | 'median-Laws E5L5' | 'median-Laws R5E5S5' |
|  | 'median-Laws W5S5' | 'skewness-Laws W5L5S5' |
|  | 'Collage_skew_MaximalCorrelationCoefficient_1_nb_4_ws_3' | 'Collage_kurt_SumAverage_1_nb_32_ws_7' |
|  |  | 'Collage_var_InformationMeasureOfCorrelation2_1_nb_16_ws_3' |
|  |  | 'median-Laws E5L5S5' |
|  |  | 'Collage_kurt_InformationMeasureOfCorrelation2_1_nb_4_ws_5' |
|  |  | 'Collage_kurt_InformationMeasureOfCorrelation2_2_nb_16_ws_7' |
|  |  | 'Collage_kurt_InformationMeasureOfCorrelation2_1_nb_16_ws_3' |
|  |  | 'median-Laws E5E5E5' |
|  |  | 'median-Laws W5E5S5' |
| Trial 4 | 'skewness-Laws R5S5' | 'skewness-Laws E5E5R5' |
|  | 'Collage_kurt_MaximalCorrelationCoefficient_1_nb_4_ws_5' | 'skewness-Laws E3S3E3' |
|  | 'Collage_skew_InformationMeasureOfCorrelation2_1_nb_8_ws_3' | 'skewness-Laws R5E5E5' |
|  | 'Collage_kurt_Contrast_1_nb_8_ws_7' | 'skewness-Haralick correlation ws=5 n=4' |
|  | 'Collage_median_MaximalCorrelationCoefficient_1_nb_32_ws_3' | 'skewness-Laws E3E3E3' |
|  | 'Collage_skew_Correlation_1_nb_16_ws_5' | 'Collage_kurt_SumAverage_1_nb_16_ws_7' |
|  | 'Collage_skew_MaximalCorrelationCoefficient_1_nb_4_ws_3' | 'Collage_kurt_MaximalCorrelationCoefficient_1_nb_4_ws_7' |
|  | 'Collage_skew_SumVariance_1_nb_8_ws_5' | 'skewness-Haralick info1 ws=5 n=64' |
|  | 'median-Laws E5L5' | 'Collage_kurt_InformationMeasureOfCorrelation2_2_nb_16_ws_7' |
|  | 'Collage_skew_Entropy_1_nb_16_ws_7' | 'skewness-Laws R5R5L5' |
|  | 'skewness-Laws S5E5' | 'Collage_var_SumEntropy_1_nb_32_ws_7' |
|  | 'skewness-Laws W5L5' | 'median-Laws R5E5L5' |
|  | 'Collage_skew_DifferenceEntropy_1_nb_4_ws_3' |  |
|  | 'Collage_var_Contrast_1_nb_8_ws_7' |  |
|  | 'Collage_kurt_SumEntropy_1_nb_16_ws_5' |  |

**Supplemental Table S2.** Detailed description of each radiomic family group.

| **Family** | **Description** | **Features** | **Parameters** | **Raw**  **features** | | **Statistics** | **Total features** | |
| --- | --- | --- | --- | --- | --- | --- | --- | --- |
|  |  |  |  | **2D** | **3D** |  | **2D** | **3D** |
| Raw | Intensity | 1 | N/A | 1 | 1 | Median  Variance  Kurtosis Skewness | 4 | 4 |
| Gray | Median, Mean, Std and Range filters | 4 | ws: 3,5, 7 | 12 | 12 |  | 48 | 48 |
| Gradient 2D | sobel x, sobel y, sobel xy, sobel yx, Gradient x, Gradient y, Gradient magnitude, Gradient dx, Gradient dy and Gradient diagonal | 10 | ws: 3 | 10 | N/A |  | 40 | N/A |
| Gradient 3D | sobel x, sobel y, sobel z, sobel xy, sobel yx, sobel xz, sobel zx, sobel yz, sobel zy, Gradient x, Gradient y, Gradient z, Gradient magnitude | 13 | ws: 3 | N/A | 13 |  | N/A | 52 |
| Laws 2D | 34 filters | 34 | N/A | 34 | N/A |  | 136 | N/A |
| Laws 3D | 152 filters in 3D | 152 | N/A | N/A | 152 |  | N/A | 608 |
| CoLiAGe  2D | Angular second moment, Contrast, Correlation, Sum sqrt var, Sum var, Sum average, Sum entropy, Entropy, Diff variance, Diff entropy, Info Corr 1, Info Corr 2 and Maximal correlation coefficient | 13   (1 SVD orientation) | ws: 3, 5, 7   # of bins: 4,8,16,32,64 | 195 | N/A |  | 780 | N/A |
| CoLiAGe  3D |  | 13  (2 SVD orientations) |  | N/A | 390 |  | N/A | 1560 |
| Haralick | Entropy, energy, Inertia, idm, Correlation, Info1, Info2, sum av, sum var, sum entropy, diff av, diff var, diff entropy | 13 | ws: 3, 5, 7   # of bins: 4,8,16,32,64 | 195 | 195 |  | 780 | 780 |
